# Supplementary material for: Windows of opportunity for predicting seasonal climate extremes highlighted by the Pakistan floods of 2022
Source: Nat Commun. 2023 Oct 17;14:6544. doi: 10.1038/s41467-023-42377-1 (PMC10582174; doi:10.1038/s41467-023-42377-1)
Supplement: Supplementary file 1 — Supplementary information [file 41467_2023_42377_MOESM1_ESM.pdf]

Windows of opportunity for predicting seasonal climate extremes: Pakistan floods of 2022

SUPPLEMENTARY INFORMATION

Nick Dunstone[1*], Doug M. Smith[1], Steven C. Hardiman[1], Paul Davies[1], Sarah Ineson[1], Shipra Jain[3], Chris Kent[1], Gill Martin[1], Adam A. Scaife[1,2]

[1]Met Office Hadley Centre, United Kingdom

[2]University of Exeter, United Kingdom

[3]Centre for Climate Research Singapore (CCRS), Singapore

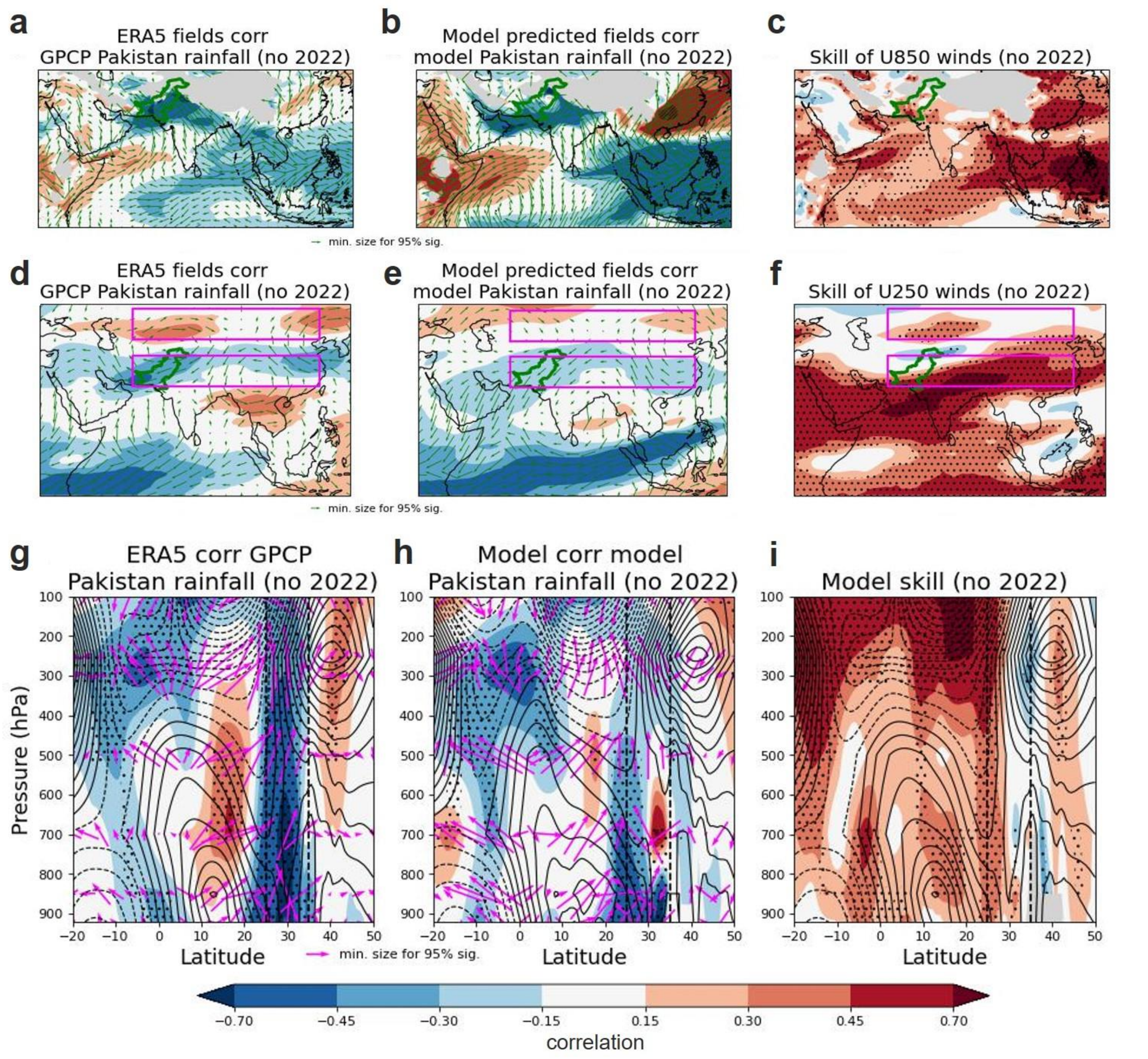

Figure S1: Circulation anomalies associated with Pakistan summer rainfall excluding summer 2022. **a-c**, as Fig. 2d-f but excluding 2022. **d-f**, as Fig. 3d-f but excluding 2022. **g-i**, as Fig. 4d-f but excluding 2022.

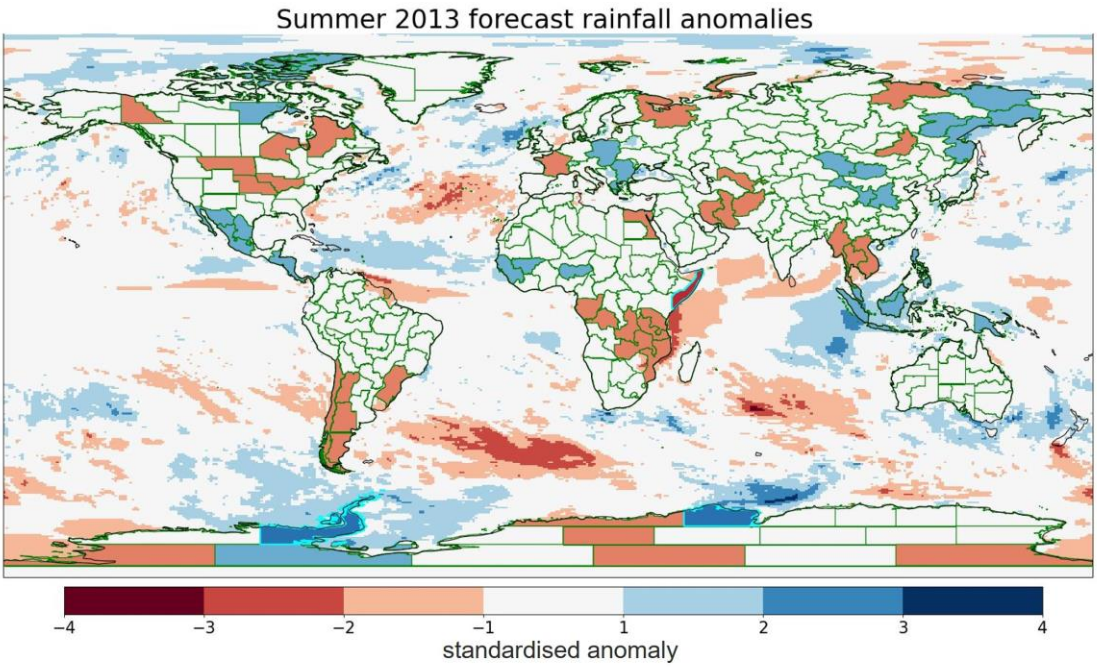

Figure S2: As Fig. 6 but for predicted standardised ensemble mean rainfall anomalies in summer 2013. This is an example of a year where very few predicted extreme rainfall regions, with only one region (Somalia) having a greater than ±2σ anomaly.

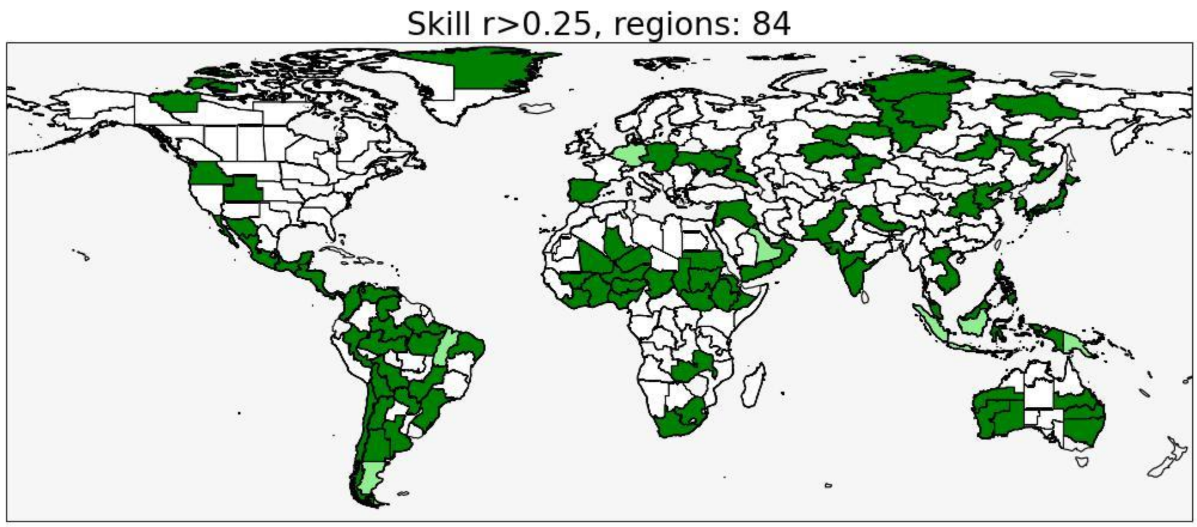

Figure S3: The 84 regions (pale and dark green) that show significant DP3 summer rainfall skill (r>0.25) over 1979-2021. Note 7 of these regions (pale green) are not used in the analysis in Fig. 7a as they do not simulate a ≥±2σ ensemble mean extreme over 1979-2022.
